# An epidemiological investigation of food allergy among children aged 3 to 6 in an urban area of Wenzhou, China

**DOI:** 10.1186/s12887-020-02115-8

**Published:** 2020-05-14

**Authors:** Huan Dai, Fangmin Wang, Like Wang, Jinyi Wan, Qiangwei Xiang, Hui Zhang, Wei Zhao, Weixi Zhang

**Affiliations:** 1 Department of Pediatric Pulmonology, The Second Affiliated Hospital and Yuying Children’s Hospital of Wenzhou Medical University, 109 Xueyuan Road, Wenzhou, 325027 Zhejiang Province China; 2 Division of Allergy and Immunology, Department of Pediatrics, Virginia Commonwealth University, Richmond, VA USA

**Keywords:** Food allergy, Preschool, Epidemiology, Prevalence

## Abstract

**Background:**

The prevalence of food allergy (FA) has increased worldwide. In China, the prevalence of FA in infants and school-aged children is well known, but the prevalence in preschool children is unknown.

**Methods:**

A total of 4151 preschool children aged 3 to 6 years in urban Wenzhou, China, were recruited for this cross-sectional study. Their parents completed a preliminary screening questionnaire, and a detailed FA questionnaire was given to parents whose children had suspected FA according to the preliminary screening. According to the results of the detailed FA questionnaires, some children underwent a skin prick test (SPT) and specific IgE (sIgE) measurement. Children with abnormal SPT and/or sIgE results who did not meet the diagnostic criteria and those with negative SPT and sIgE results whose histories strongly supported FA underwent an oral food challenge (OFC).

**Results:**

Of the 4151 children’s parents who completed the surveys, 534 (12.86%) indicated a positive medical history of FA. Among the 40 children who underwent an OFC, 24 were positive. According to SPT and sIgE measurements, 11 children were diagnosed with FA. The prevalence of FA was at least 0.84%; children who dropped out during the study were considered FA-negative. Among the 35 children with FA, the most common allergic manifestation was skin symptoms. The most common allergic foods were egg, fish and shrimp.

**Conclusions:**

The parent-reported rate of FA in preschool children in urban Wenzhou was 12.86%. The prevalence of FA was at least 0.84%. Among all cases, the most common allergic food was eggs, and the most common allergic manifestation was skin symptoms.

**Trial registration:**

NCT03974555, registered on 30 May 2019 (www.clinicaltrials.gov).

## Background

Food allergy (FA) is an adverse reaction to food that is induced by an abnormal or excessive immune response to food allergens. FA includes IgE-mediated, non–IgE-mediated (cell-mediated), or mixed (IgE and cell-mediated) pathophysiologies [1]. FA manifests as various symptoms, including skin, respiratory, digestive, and cardiovascular symptoms [2]. Following asthma and allergic rhinitis, FA has recently become another allergy epidemic [3]. Worldwide, the prevalence of FA has increased over the last 30 years, with a 6–8% prevalence rate in children [4]. FA significantly impacts the quality of life of children and their families.

The exact prevalence of FA is difficult to determine because the characteristics of FA differ among races, ages and regions; FA is associated with geographical and dietary differences and countless other unknown factors [5]. The parent-reported rate of FA has been recently increasing as indicated by the epidemiological data of FA. Studies have indicated that the self-reported rate of FA increases by 1.2% every 10 years [6]. The diagnosis of FA involves obtaining histories (including diet records), a physical examination, a skin prick test (SPT), specific IgE (sIgE) measurement, trial elimination diets and food challenges. The World Allergy Organization (WAO) found that 10% of countries maintain FA prevalence data based on oral food challenges (OFCs) [7]**.** According to a study in the United States, in which data were collected by questionnaires and sIgE measurements, the prevalence of FA has increased to 18% [8]. A study in Australia showed that approximately 10% of 12-month-old children were positive for FA, which was confirmed by an OFC [9]. In Thailand, the self-reported rate of FA in preschool children was 9.3%, and the prevalence of FA confirmed by OFC was at least 1.11% (95% CI, 0.41–2.98%) [10]. In Japan, the prevalence was difficult to obtain, but approximately 350,000 children were diagnosed with FA by doctors [11].

The epidemiology of FA has been extensively studied in other countries and regions, but it has rarely been studied in mainland China. The prevalence of FA and the self-reported rates of FA vary widely. Chen et al. [12] reported that the prevalence rates of FA in children younger than 2 years old in Chongqing, Zhuhai and Hangzhou were 7.3, 5.8 and 5.6%, respectively, and a common allergen was eggs. In Guangzhou, a study showed that the self-reported rate of FA in school-age children was 14.6%, and the rate confirmed by SPT and sIgE measurement was 0.31%. Common allergens included shrimp and crab [13].

In China, the FA prevalence in infants and school-aged children is well known, but the exact prevalence of FA in preschool children is unknown. At 3 to 6 years of age, preschool children are engaging in social experiences for the first time, and both their parents and kindergarten teachers are highly concerned about FA. In the most severe case, FA can lead to anaphylaxis and might result in death within minutes, although it is rare. Therefore, we conducted an epidemiological survey of FA among children aged 3 to 6 years in an urban area of Wenzhou, which will have important scientific and social value.

## Methods

### Study design

**Fig. 1:**
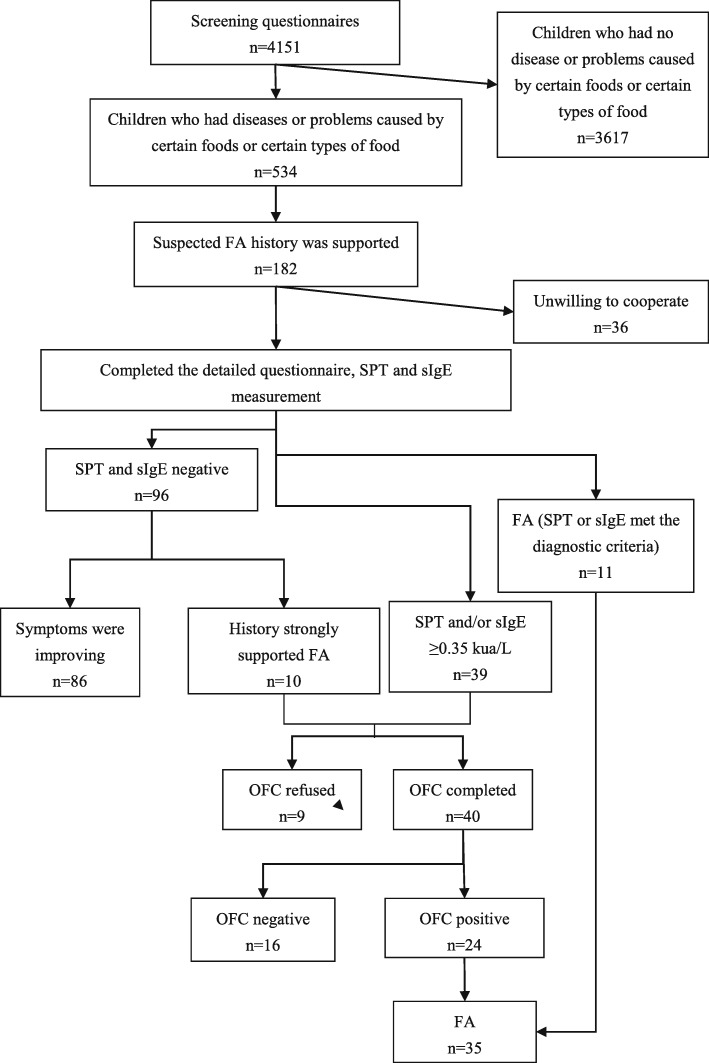
The flow chart of the food allergy epidemiological investigation

Figure 1 shows the flow chart of the study process. This design has been registered in the clinical trials database (ID: NCT03974555). In this study, we selected a random sample of children aged 3 to 6 years from 11 kindergartens in an urban area of Wenzhou, including the Lucheng district, Ouhai district and Longwan district. The study was approved by the medical ethical committee of The Second Affiliated Hospital and Yuying Children’s Hospital of Wenzhou Medical University (Project number: LCKY2018–06), and written informed consent was obtained from the parent or legal guardian of the children.

### FA questionnaires

All the participating subjects’ parents or guardians were asked to complete the FA screening questionnaire via WeChat. Some parents indicated that their children had diseases or problems caused by certain foods or certain types of food. Researchers ensured that the histories were correct by telephone interviews. Then, the parents were asked to complete the detailed EuroPrevall FA questionnaire [14], and their children underwent medical testing. All the screening questionnaires and detailed questionnaires were checked for completeness by the researchers.

### SPT and sIgE measurement

Children who were suspected of having FA underwent SPTs and sIgE measurements after their parents completed the detailed questionnaires. Seventeen allergens including milk, egg white, egg yolk, shrimp, crab, wheat, mackerel, perch, codfish, peanut, cashew, soybean, peach, pineapple, mango, orange and kiwi fruit (Greer Laboratories Inc., America) were used for the SPT. Nine allergens including milk, egg white, shrimp, crab, soybean, peanut, wheat, a mixed nut group and a mixed food group (ALK-Abelló, Denmark) were measured by ImmunoCAP (Phadia25, Phadia AB, Sweden). The mixed food group included egg white, milk, wheat, fish, peanut and soybean.

The SPT results were considered positive if the mean wheal diameter was 3 mm or greater after subtraction of the saline control. Additionally, sIgE measurement results greater than 0.35 kIU/L were considered positive.

### OFC

Children with abnormal SPT and/or sIgE results who did not meet the diagnostic criteria and those with negative SPT and sIgE results with histories strongly suggestive of FA underwent an OFC. Discontinuation of some medications that may interfere with the OFC may be needed (Table 1) [15].

**Table 1:** Guidelines for discontinuation of medications that might interfere with interpretation of OFC

Medication	Last dose before OFC
Oral antihistamines	3-10d
Cetirizine	5-7d
Loratadine	7d
Oral H2 receptor antagonist	12 h
Oral long-acting β2-agonist	24 h
Theophylline (liquid)	24 h
Leukotriene antagonist	24 h
Oral/intramuscular/intravenous steroids	3d-2wk

The OFCs were conducted in the hospital under the supervision of an allergist with close observation of any adverse signs and symptoms, and emergency medicine was prepared because OFC may lead to severe anaphylaxis in some cases. The total amounts of challenge foods administered during the gradually escalating OFC were 8 to 10 g of dry food, 16 to 20 g of meat or fish, and 100 mL of wet food. Typically, 0.1 to 1% of the total challenge food was initially administered. The OFC was divided into 4–9 increments, from a minimum dose to a maximum dose; each challenge was administered at a 30-min interval until objective mild symptoms were elicited [15]. Vital signs were monitored before and after the OFC.

### Statistical methods

Data are presented as the rates and means±standard deviations. Data were compared using chi-square tests. *P* values < 0.05 were considered statistically significant. All statistical analyses were performed using SPSS software.

## Results

The researchers collected 4151 questionnaires, analyzed all information and conducted further investigations and tests as necessary. Table 2 shows the details of parent-reported FAs and confirmed FAs in this study.

**Table 2:** The details of parent-reported FA and FA in this study

Variable	Total sample	Parent-reported FA(n)	FA (n)
All	4151	534	35
Sex			
Male	2174	284	24
Female	1977	250	11
Age			
≥ 3 to < 4	1423	181	15
≥ 4 to < 5	1348	193	10
≥ 5 to < 6	1374	159	10
≥ 6 to < 7	6	1	0
Clinical manifestation			
Skin rash and pruritus		359	31
Itching, tingling or swelling in mouth, lips or throat		160	20
Diarrhoea or vomiting (excluding food poisoning)		123	12
Sneezing, runny nose or stuffy nose		89	8
Cough		39	2
Eyes rubefaction, pain or lacrimation		37	8
Dyspnoea		18	3
Others		45	4
Food			
Milk		81	4
Egg		111	13
Shrimp		102	5
Crab		81	4
Mango		82	0
Fish		56	9
Peanut		10	2
Others		96	0
Allergic history			
Eczema	2035	414	30
Rhinitis	1015	231	17
Wheezing	368	92	8
Diagnosed with FA previously	452	281	25

### FA questionnaires and parent-reported FA rate

A total of 4151 children’s parents completed the FA screening questionnaires, resulting in response rates of 52.37 and 47.63% for males and females, respectively. The average age was 3.99 ± 0.824 years. In total, 534 parents indicated that their children had diseases or problems caused by certain foods or certain types of food; therefore, the parent-reported rate of FA was 12.86%. There was no significant difference in the parent-reported rate of FA between the sexes (males 284 vs. females 250) in children aged 3 to 6 years in an urban area of Wenzhou (*P*>0.05). Among the parent-reported cases, the most common allergic food was eggs, and the most common allergic manifestation was skin symptoms.

After telephoning 534 parents or guardians, 352 children were excluded. In one of the 352 children, the symptoms were not caused by food, and another child gradually became food tolerant. A total of 182 children completed medical tests, and their parents completed the detailed FA questionnaire, but 36 dropped out before the end of the study.

### SPT and sIgE measurement

SPTs and sIgE measurements were conducted in 146 children. Among the 17 allergens included in the SPT, the allergen with the highest positive rate was shrimp, accounting for 13.01%, followed by crab (12.33%), eggs (11.64%), fish (10.96%) and milk (4.79%). Among the 9 allergens included in the sIgE measurement, the most common allergen was egg white, accounting for 13.70%, followed by milk (13.01%), shrimp (8.90%), the mixed food group (4.79%) and crab (4.11%).

According to the results of the SPTs and sIgE measurements, 50 children were positive, and 11 of the 50 children were diagnosed with FA. A total of 96 children were negative according to these two tests.

### OFC and the prevalence of FA

Thirty-nine children with a positive SPT and/or sIgE measurements had abnormal test results but did not meet the FA diagnostic criteria; moreover, 10 children were negative according to the SPT and sIgE measurements, but their histories strongly supported FA. These 49 children were scheduled for an OFC, but the parents or guardians of nine children refused the OFC. Among the 40 children who underwent the OFC, 24 had positive results. Therefore, in this study, 35 children were diagnosed with FA. The prevalence of FA among children aged 3 to 6 years in an urban area of Wenzhou was at least 0.84% because children who were very likely to have FA and who dropped out during the study were considered negative for FA. In FA-positive children, the most common allergic manifestation was skin symptoms, accounting for 88.57%. The six leading causes of FA were eggs (37.14%), fish (25.71%), shrimp (14.29%), milk (11.43%), crab (11.43%) and peanuts (5.71%).

## Discussion

In this epidemiological investigation, the parent-reported FA rate of preschool children aged 3 to 6 years in an urban area of Wenzhou was 12.86%, and the prevalence of FA was at least 0.84%. Assuming that the likelihood of confirmed FA was equal among the participants and dropouts, the adjusted estimated prevalence of FA in children was approximately 1.36%. This study also indicated that common allergens in preschool children included eggs, fish, shrimp, milk and crab. Skin symptoms were the most common allergic manifestation**.**

Among Asian countries, the self-reported FA rate in preschool children in Thailand was 9.3% [10]; a study in Vietnam showed that among children aged 2 to 6 in Hue, the self-reported FA was 9.8%, and in Tien Giang the rate was 7.9% [16]; additionally, a multi-city study in China showed that the self-reported FA rate in children aged 3 to 5 years was 6.65% [17]. Parents may exaggerate the reaction caused by FA because of their anxiety. A disparity was observed between the parent-reported rate and the prevalence of FA among different regions; the prevalence of FA was lower than the parent/self-reported rate. In some developed countries, the FA prevalence in infants was 10% [7]. A systematic review showed that the FA prevalence rates were similar in children aged 0 to17 years and adults, at approximately 0.9% (95% CI: 0.8–1.1%) [18]. The prevalence of FA in preschool children in Thailand was ≥1.11% (95% CI: 0.41–2.98%) [10]. Children under 3 years old in China had a prevalence rate of 3.5 to 7.3% [12, 19]. A study in Guangzhou, China, showed that the FA prevalence in children aged 7 to 12 years was 0.31% [13]; this result was confirmed by SPTs and sIgE measurements. The FA prevalence was higher in this study (0.58%) than in the study in Guangzhou. To a certain extent, the FA prevalence in the urban area of Wenzhou is not lower than the prevalence in Guangzhou. The prevalence of FA in this investigation (0.84%) was between that of infants and school-aged children in the abovementioned areas. Among the children with FA, 5.71% were positive for multiple allergens; this result was significantly lower than the parent-reported rate of 57.30%. A possible reason is that children may have developed a tolerance to some types of food, such as milk. One study showed that 87% of children who had milk allergies as infants tolerated milk at 3 years of age [20].

In this study, 534 children had parent-reported FA, and 281 of the 534 children had been diagnosed with FA by doctors, but only 35 children were confirmed to have FA. The number of children who were confirmed to have FA was significantly lower than the number of children who were previously diagnosed with FA by doctors, consistent with the above studies. On the one hand, the parents or guardians in this study had a high level of education and may have had excessive knowledge of FA. They may have interpreted all food-related adverse reactions as FA. In addition, some types of food will gradually become tolerated as children age. On the other hand, the diagnosis of FA still faces the challenges mentioned above, and some parents or guardians refused to complete the detailed questionnaire and consent to the corresponding laboratory tests. To some degree, this result shows the rate of overdiagnosis of FA. The greatest source of misdiagnosis of FA might be the lack of appreciation that a positive test result does not indicate an allergy [1]. Thirty-five children in the study were diagnosed, but only 71.43% were ever diagnosed by doctors. This result also shows a deficiency in FA diagnosis and indicates that FA need to be diagnosed by a professional allergist. A medical history is crucial for the diagnosis of FA. Several diagnostic methods exist for FA, including SPTs, sIgE measurements, and OFC.

A study showed that sIgE measurements were more sensitive than SPTs in infants; however, in black race, SPTs tended to be more accurate [21, 22]. DunnGalvin et al. [23] used six indices, namely, age, sex, symptoms, SPT results, total IgE (tIgE) results and sIgE results, to predict the clinical diagnosis. They indicated that these six indices had higher sensitivity and specificity than an SPT, sIgE measurement or both. Although the gold standard for FA diagnosis is a double-blinded placebo-controlled food challenge (DBPCFC), in clinical work, an OFC is sufficient to diagnose FA in children. However, an OFC is not commonly performed because of some limitations. SPTs and sIgE measurements are commonly used to diagnose FA, but these results have higher sensitivity, resulting in false positives; therefore, the medical history should be considered along with these results [1]. When clinical history is supported by results of SPTs and/or sIgE measurements at a 95% PPV, FA is assumed without need for an OFC (Table 3) [15]. There were some children in the study who had a FA history and a positive SPT and (or) sIgE measurement, but the results did not reach the standard. A portion of children had a negative SPT and sIgE measurement, but their history strongly supported FA. These situations require an OFC.

**Table 3:** The cutoff value of SPT and sIgE measurement for children over 2 years old

	sIgE (kua/L)	SPT (mm)
Milk	≥15	≥8
Egg	≥7	≥7
Peanut	≥14	≥8
Fish	≥20	

In this study, eggs, fish and shrimp were common allergens in children with FA, while eggs, shrimp and mango were common in parent-reported data. Due to differences in dietary habits, peanuts and wheat have high allergic rates in the United States. In North America and Northern Europe, allergic reactions to fish and shellfish are common, and allergic reactions to Prunoideae fruits are common in the Mediterranean region [24]. In most parts of Asia (China, Korea and some South East Asian countries), egg allergy predominates over cow’s milk in children younger than 5 years. Shellfish (crustaceans and mollusks) allergy is the most common food allergy in older children and adults in Asia [25]. Le et al. [16] found that crustaceans are the predominant allergy-inducing food among children aged 2 to 6 in Vietnam. Allergens varied by region even within China. Liu et al. [26] found that the most common self-reported food allergens were eggs among children aged 0–12 months, shrimp among children aged 13–24 months, and fish among children aged 25 to 36 months. In Beijing the main allergen was fruit among children aged 0–14 months [27]. In Guangzhou, shrimp and crab were the most common allergy-inducing foods [12]. Among the patients diagnosed by OFC, the most common allergen in preschool children in Thailand was shrimp [10]. In Chongqing, China, eggs and milk were common allergens among children aged 0 to 1 year with a challenge-proven FA [28]. In Shanghai, the main allergens were eggs, milk, shrimp and fish. Egg allergy was common in children younger than 3 years, and shrimp allergy was common in children older than 3 years [29]. Studies have shown that egg allergy resolves by half at a median age of 74 months, while milk allergy resolves by half at 66 months [30, 31].

The strengths of this study were the large population-based dataset and the administration of an OFC. This study revealed the parent-reported rate, prevalence rate and clinical features of FA. However, there were some limitations of this study. First, selection bias such as nonresponse bias is unavoidable. Second, the loss to follow-up and the limited allergen test spectrum may have led to some degree of other bias.

## Conclusions

In this study, the parent-reported rate of FA in preschool children in urban Wenzhou was 12.86%. The prevalence of FA was at least 0.84%. Among all cases, the most common allergic food was eggs, and the most common allergic manifestation was skin symptoms. These results decreased the paucity of FA data in preschool children in China. Further investigations are necessary to explore cross-reactivities between different allergens. Additionally children with FA need to be monitored to improve allergy management. Thus, we are currently working towards these goals.

## Data Availability

The datasets generated during and/or analyzed during the current study are not publicly available due to specific restrictions from the ethics committee, but are available from the corresponding author on reasonable request.

## Abbreviations

FA: Food allergy
sIgE: Specific IgE
OFC: Oral food challenge
SPT: Skin prick test
tIgE: Total IgE
DBPCFC: Double-blinded placebo-controlled food challenge
PPV: Positive predictive value
